# Phenotypic Variability in the Coccolithophore *Emiliania huxleyi*

**DOI:** 10.1371/journal.pone.0157697

**Published:** 2016-06-27

**Authors:** Sonia Blanco-Ameijeiras, Mario Lebrato, Heather M. Stoll, Debora Iglesias-Rodriguez, Marius N. Müller, Ana Méndez-Vicente, Andreas Oschlies

**Affiliations:** 1 National Oceanography Centre, Southampton, University of Southampton, European Way, SO14 3ZH Southampton, United Kingdom; 2 Kiel University (CAU), Christian-Albrechts-Platz 4, 24118, Kiel, Germany; 3 Department of Geology, University of Oviedo, Arias de Velasco s/n, 33005 Oviedo, Spain; 4 Department of Ecology, Evolution and Marine Biology, University of California Santa Barbara, Santa Barbara, California, 93106, United States of America; 5 Oceanographic Institute at the University of São Paulo, Praça do Oceanográfico 191, São Paulo, SP 05508-120, Brazil; 6 Helmholtz Centre for Ocean Research Kiel (GEOMAR), Düsternbrooker Weg 20, 24105 Kiel, Germany; Universidad Miguel Hernandez, SPAIN

## Abstract

Coccolithophores are a vital part of oceanic phytoplankton assemblages that produce organic matter and calcium carbonate (CaCO_3_) containing traces of other elements (i.e. Sr and Mg). Their associated carbon export from the euphotic zone to the oceans' interior plays a crucial role in CO_2_ feedback mechanisms and biogeochemical cycles. The coccolithophore *Emiliania huxleyi* has been widely studied as a model organism to understand physiological, biogeochemical, and ecological processes in marine sciences. Here, we show the inter-strain variability in physiological and biogeochemical traits in 13 strains of *E*. *huxleyi* from various biogeographical provinces obtained from culture collections commonly used in the literature. Our results demonstrate that inter-strain genetic variability has greater potential to induce larger phenotypic differences than the phenotypic plasticity of single strains cultured under a broad range of variable environmental conditions. The range of variation found in physiological parameters and calcite Sr:Ca highlights the need to reconsider phenotypic variability in paleoproxy calibrations and model parameterizations to adequately translate findings from single strain laboratory experiments to the real ocean.

## Introduction

The coccolithophore species *Emiliania huxleyi* belongs to a group of unicellular photosynthetic protists covered by plates of calcite (CaCO_3_) termed coccoliths [1]. Coccolithophores are major producers of CaCO_3_ [2] and key players in the Earth's climate system [3]. They contribute to the biological carbon pump via the combined effects of calcification, photosynthesis, and the downward transport of particulate material to the oceans' interior [4]. Although there are numerous extant coccolithophore species, *E*. *huxleyi* is used as a model species in many physiological [5], ecological [6], oceanographic [7], paleoceanographic [8], and modelling studies [9] because of its global importance and the ease to culture it under a broad range of environmental conditions [10].

*Emiliania huxleyi* harbors a genome constituted by core genes plus genes distributed variably amongst strains, which supports a considerable intra-species variability [11]. This enables *E*. *huxleyi* to form large seasonal blooms in temperate waters and subpolar regions under a wide variety of environmental conditions [12]. Based on the morphological identity of the coccoliths, *E*. *huxleyi* is separated in seven morphotypes [13], although the relationship between geographic origin and morphotype genetic distance is unclear [14]. Within the species concept of *E*. *huxleyi*, numerous diploid non-calcifying cells have been identified which lost the ability to form calcified scales following prolonged culture [10, 15] or unfavorable experimental conditions [16]. Taking into account the morphological diversity found within the species concept of *E*. *huxleyi* a high physiological variability is expected.

The cosmopolitan distribution and bloom-forming capacity of *E*. *huxleyi* [12], its distinct optical features [17] and sinking properties [18, 19] make *E*. *huxleyi* a suitable model organism for parameterization studies in Earth System modelling. The biogeochemical signature of *E*. *huxleyi*'s coccoliths is used to develop paleoproxies because of their abundant fossil record starting in the Quaternary [20, 21]. The abundance of trace elements replacing Ca ions in calcite, such as strontium (Sr) and magnesium (Mg) is driven by thermodynamic, kinetic, and biological factors, facilitating the development of paleoproxies based on the ratio between the element of interest and calcium. The Sr:Ca ratio is used to estimate coccolithophore productivity [22], and the Mg:Ca ratio is used to reconstruct seawater temperature [23, 24] and track CO_2_ [25].Recently, the incorporation of boron (B) in the calcite has been related to seawater pH [26]. However, phenotypic variability in coccolith elemental ratios is virtually impossible to address in sediment samples because only morphotypes can be differentiated.

In this study we characterize intra-species phenotypic variation and its implications in 13 strains of *E*. *huxleyi* measuring physiological parameters (growth rate, particulate organic and inorganic carbon content [POC and PIC], particulate inorganic: organic carbon [PIC:POC],particulate carbon:nitrogen [C:N]) and coccolith geochemistry (Sr:Ca and Mg:Ca ratios). The high variability observed in our results highlights the importance of account for inter-strain variability when using *E*. *huxleyi* as a model organism in marine studies ranging from experimental work to past ocean reconstructions and model parameterizations.

## Material and Methods

### Experimental work

Monoclonal cultures of 13 *E*. *huxleyi* strains were obtained from the Provasoli-Guillard National Center for Marine Algae and Microbiota (NCMA), the Roscoff Culture Collection (RCC), the Algobank-Caen and the Microalgal Culture Collection and the Plymouth Culture Collection of Marine Algae. Nine coccolith-bearing strains, including three morphotypes (A, B and R; see Table 1), and four non-calcifying strains from various latitudes were grown in semi continuous batch cultures under identical conditions (Table 1). Strain morphotype identification followed previously published studies [15, 27, 28, 29]. Cultures were maintained at 19.15 ±0.75°C (Table 1), with a 12:12h light:dark cycle under light irradiance of 126.03 ±8.96 μmol quanta m^-2^ s^-1^ supplied by cool-white fluorescent lamps (Osram LUMILUX) and determined using a LI-COR (LI-189) Quantum Sensor (Q 39308). The cultures were daily agitated by manual swirling. Culture medium was prepared from coastal Atlantic Ocean filter-sterilized (0.22 μm) natural seawater. Salinity was determined using a refractometer. Filtered seawater was enriched with 91.4 μM of NaNO_3_, 5.3 μM of NaH_2_PO_4_ (Table 1) [30–32], and trace metals and vitamins were added according to f/2 medium [33]. Triplicate flasks containing medium were inoculated with cultures in exponential growth phase pre-acclimated for a minimum of 8 generations. Cells were inoculated at ~100 cell ml^-1^ and harvested at cell densities of 28,000 ±5,000 cell ml^-1^ to minimize changes in carbonate chemistry and nutrient availability that could potentially lead to CO_2_ and/or nutrient limitation.

**Table 1:** Origin of the *Emiliania huxleyi* strains and environmental culture conditions.

Strain^1^	Isolation	Lat.	Long.	Morphotype^2^	L:D cycle	Temperature (°C)	Salinity	DIN (μM)	DIP (μM)	Irradiance (μmol quanta m^2^ s^-1^)	Reference
CCMP370* (370*)	E. Paasche (1959)	59.83	10	un [29]	12:12	19.6 ± 0.1	35.0 ± 0.0	89.6	4.12	122.40 ± 5.83	This study
CCMP2758* (2758*)	R. Waters	50.3	-145.58	n/a	12:12	19.4 ± 0.1	35.0 ± 0.0	97.9	4.19	127.29 ± 6.99	This study
B92/11 *(B11)*	J. C. Green (1992)	46.96	5.28	A [15]	12:12	18.5 ± 0.1	36.5 ± 0.1	93.5	4.09	119.86 ± 1.99	This study
M181CCMP88E *(88E)*	R. Selvin (1988)	43	-68	A [15]	12:12	19.4 ± 0.1	35.7 ± 0.2	92.0	3.79	125.86 ± 6.94	This study
AC474 *(474)*	I. Probert (1998)	41.1	3.38	n/a	12:12	19.6 ± 0.0	35.1 ± 0.1	91.1	4.71	117.08 ± 5.94	This study
RCC1258 *(1258)*	I. Probert (1998)	40.58	-10	B	12:12	19.6 ± 0.1	35.0 ± 0.1	97.3	2.30	127.85 ± 3.63	This study
M184CCMP1A1 *(1A1)*	B. Palenik (1987)	32	-62	A [29]	12:12	19.5 ± 0.2	36.3 ± 0.1	89.5	3.26	124.50 ± 5.56	This study
CCMP2090* (2090*)	J. Sexton (1991)	-2.82	-83.01	n/a	12:12	19.5 ± 0.2	34.9 ± 0.1	90.1	4.12	118.78 ± 4.85	This study
CCMP1280* (1280*)	F. Valois (1985)	-12	-35	n/a	12:12	18.9 ± 0.1	34.9 ± 0.1	94.6	4.05	120.12 ± 3.75	This study
South Africa *(SA)*	R. Pienaar (1983)	-29.85	31.05	A [15]	12:12	19.2 ± 0.1	35.0 ± 0.0	98.6	4.27	121.02 ± 5.91	This study
RCC1212 *(1212)*	I. Probert (2000)	-34.46	17.3	B [27]	12:12	19.7 ± 0.0	35.0 ± 0.1	90.1	1.75	134.99 ± 1.63	This study
NZEH *(NZEH)*	L. Rhodes (1992)	-46.96	168.08	R [27]	12:12	18.4 ± 0.1	35.3 ± 0.1	95.5	3.94	120.94 ± 4.36	This study
AC472 *(472)*	n/a	-48.3	169.83	R [27]	12:12	19.9 ± 0.1	35.1 ± 0.1	89.2	6.31	120.55 ± 4.77	This study
***Average***					***12*:*12***	***19*.*3 ± 0*.*4***	***35*.*3 ± 0*.*5***	***93*.*0***	***3*.*9***	***123*.*2 ± 4*.*8***	**This study**
RCC1238	I. Probert (2005)	n/a	n/a	A [27]	16:8	20.0	32.0	100	6.25	400	[27]
CCMP374	Skinner (1990)	n/a	n/a	n/a	n/a	20.0	35.0	100	6.00	150	[30]
AC481	I. Probert	n/a	n/a	n/a	14:10	18.0	35.6	32	1.00	150	[31]
RCC1212	I. Probert (2000)	n/a	n/a	B [27]	16:8	20.0	32.0	100	6.25	400	[27]
NZEH	L. Rhodes (1992)	n/a	n/a	R [27]	12:12	19.0	34.0	100	6.24	150	[32]
NZEH	L. Rhodes (1992)	n/a	n/a	R [27]	n/a	20.0	35.0	100	6.00	150	[30]

^(1)^ Strain name in backets and italics correspond to the abreviations used in Figs 3 and 4.

*Indicates the non-calcifying strains.

^(2)^ Morphotypes classification extracted from the literature and references.

(un) undetermined. (n/a) no data available.

Cell density, pH, salinity and irradiance were monitored during the incubations. Daily *in-situ* pH_total_ and temperature were determined on sub-samples using a pH-meter with a temperature probe (EUTECH EcoScan—pH/mV/°C) (S1 Table). During the experiments the medium carbon chemistry was measured for each replicate flask. Both, daily monitoring and final harvesting were performed at the same time of the day within a 3–5 h interval after the start of the light phase.

### Inorganic elemental chemistry

The seawater carbonate system parameters were calculated from temperature, salinity, and the concentration of dissolved inorganic carbon (DIC), total alkalinity (TA) and phosphate using the software CO2SYS [34] (S1 Table). Total alkalinity and DIC were determined using the (Versatile INstrument for the Determination of Tritation Alkalinity) VINDTA, calibrated with Certified Reference Material (CRM) for oceanic CO_2_ measurements, Marine Physics Laboratory of Scripps Institute of Oceanography, University of San Diego, following Mintrop [35]. Precisions for TA and DIC were ±7.45 μmol kg^-1^ and ±4.7 μmol kg^-1^, respectively.

Dissolved inorganic nitrate (DIN) and dissolved inorganic phosphate (DIP) were determined according to Hansen and Koroleff [36] using a HITACHI U-2000 Spectrophotometer at the GEOMAR, Kiel, Germany. The precisions for DIN and DIP were ±0.1 μM and ±0.02 μM, respectively.

In order to measure calcite Sr:Ca and Mg:Ca ratios cells were concentrated by centrifugation of ~1.5 l of culture at 1970 × g for 20 min using a Hettich ROTANTA 460RS Centrifuge, and subsequent freeze drying of the pellet. Before analysis, the samples were treated to remove organic-Mg cellular phases with hydroxylamine-hydrochloride (NH_2_OH·HCl + NH_4_OH) followed by three consecutive oxidation steps (using H_2_O_2_) according to Blanco-Ameijeiras *et al*. [37]. The samples were dissolved in 50 μl of ultrapure 2% HNO_3_ and diluted to 500 μl with Milli-Q. Elemental composition of the samples was determined using the Thermo *i*CAP 6300 Series ICP Spectrometer, at the Geology Faculty of the University of Oviedo (Spain). All samples were diluted to a common Ca concentration, seeking the highest possible value within the range of standard calibration solutions (Ca = 15, 50, 100 ppm). Calibrations were performed with multi-element standards offline using the intensity ratio method described in de Villiers *et al*. [38].

Seawater Sr:Ca and Mg:Ca ratios were determined separately by the method of standards addition in culture medium samples filtered through 0.22 μm, diluted to 1:200 and 1:10 respectively and measured with a Thermo *i*Cap 6300 Series ICP spectrometer as described above. The partition coefficients of Sr (D_Sr_) and Mg (D_Mg_) between coccolith calcite and seawater were calculated following Eq 1, where *x* is the trace element of interest.

$$D_{x}=\frac{{\left(\frac{x}{Ca}\right)}_{calcite}}{{\left(\frac{x}{Ca}\right)}_{seawater}}$$ (1)

### Growth rates and cell morphometrics

Cells were counted using a ZEISS Axioskop 40 light microscope with 200× magnification in a Neubauer haemocytometer (0.1 mm depth; 0.0025 mm^3^). Growth rate (μ) was calculated following Eq 2, where c_0_ and c_1_ are the cell concentrations at the beginning and at the end of the experiment, respectively, and Δ*t* is the period of incubation in days. Growth rate was determined as the average counts on triplicate measurements.

$$\mu =\frac{\left(Log_{e}\;C_{1}-Log_{e}\;C_{0}\right)}{\Delta t}$$ (2)

Determination of coccosphere and cell volume was performed on a Beckman Coulter Multisizer III, fitted with an aperture tube orifice diameter of 70 μm. Analyses were performed with the program MULTI 32 program (Beckman Coulter). Coccospheres were decalcified to determine the cell volume by adding HCl to culture aliquots until pH 5.0 was reached, which results in coccolith dissolution. After two minutes, the pH was rapidly readjusted to its original value in the culture by adding of 100 mM NaOH, following a modified version of the method described by Linschooten *et al*. [39]. Cell integrity was checked with the optical microscope with 400× magnification.

### Particulate carbon analysis

Samples for total particulate carbon (TPC), particulate organic carbon (POC), total particulate nitrogen (TPN), and particulate organic nitrogen (PON) were prepared by filtering aliquots onto pre-combusted 25 mm GF/F filters, followed by storage at -20°C until required for subsequent analysis. The samples for POC and PON analysis were fumed with sulfurous acid for 24 h [40] to remove particulate inorganic carbon (PIC) from the filters. The filters were dried at 60°C for 24 h, packaged in pre-combusted aluminum foil [41], and analyzed on a Thermo Finnegan flash EA1112 elemental analyzer using acetanilide as the calibration standard. PIC was calculated as the subtraction of POC from TPC. Inorganic and organic carbon production (Prod) were calculated according to Eq 3 and expressed in pg C cell^-1^ d^-1^.

Prod= μ  × cellular carbon  content (3)

In addition, calcium (from the PIC) was measured using a Thermo *i*Cap 6300 Series ICP Spectrometer to determine PIC assuming that Ca concentration in organic phases is negligible. The two methods to measure PIC were compared to each other because both are widely used in the literature and results are often assumed to be comparable (see details and discussion in S1 Notes, S1 Fig).

### Data analyses

Averaged values are given by the statistical mean and the standard deviation (SD) of three replicate samples per treatment. One-way analysis of variance (ANOVA) was used to test that the differences in biogeochemical parameters (coccosphere volume, cell volume, growth rate, particulate carbon and nitrogen cell quotas, particulate carbon production, stoichiometric ratios and D_Sr_) between strains were statistically different. Correlation of the biogeochemical parameters was determined using the Pearson product-moment correlation coefficient (r). Statistical analyses were performed using SigmaPlot (Systat Software, San Jose, CA).

## Results

### Culture conditions

The average seawater pH_total_ for all the strains was 7.97 ± 0.04, *p*CO_2_ was 479.82 ± 37.96 μatm, total alkalinity (TA) was 2272.40 ± 18.04 μmol kg^-1^, dissolved inorganic carbon (DIC) was 2055.39 ± 13.16 μmol kg^-1^ and Ω-Calcite was 3.75 ± 0.25 (S1 Table). The shift in DIC due to cells consumption and production of inorganic carbon was <2.5%. Average nitrate concentration was 93.0 ± 3.4 μM and average phosphate concentration was 3.9 ± 1.1 μM (Table 1). Irradiance during the incubation oscillated between 117.08 ±5.94 and 134.99 ±1.63 μmol quanta m^2^ s^-1^ and the average temperature during the incubation was 19.32 ±0.45°C.

### Physiological parameters

#### Cellular volume

**Fig 1:**
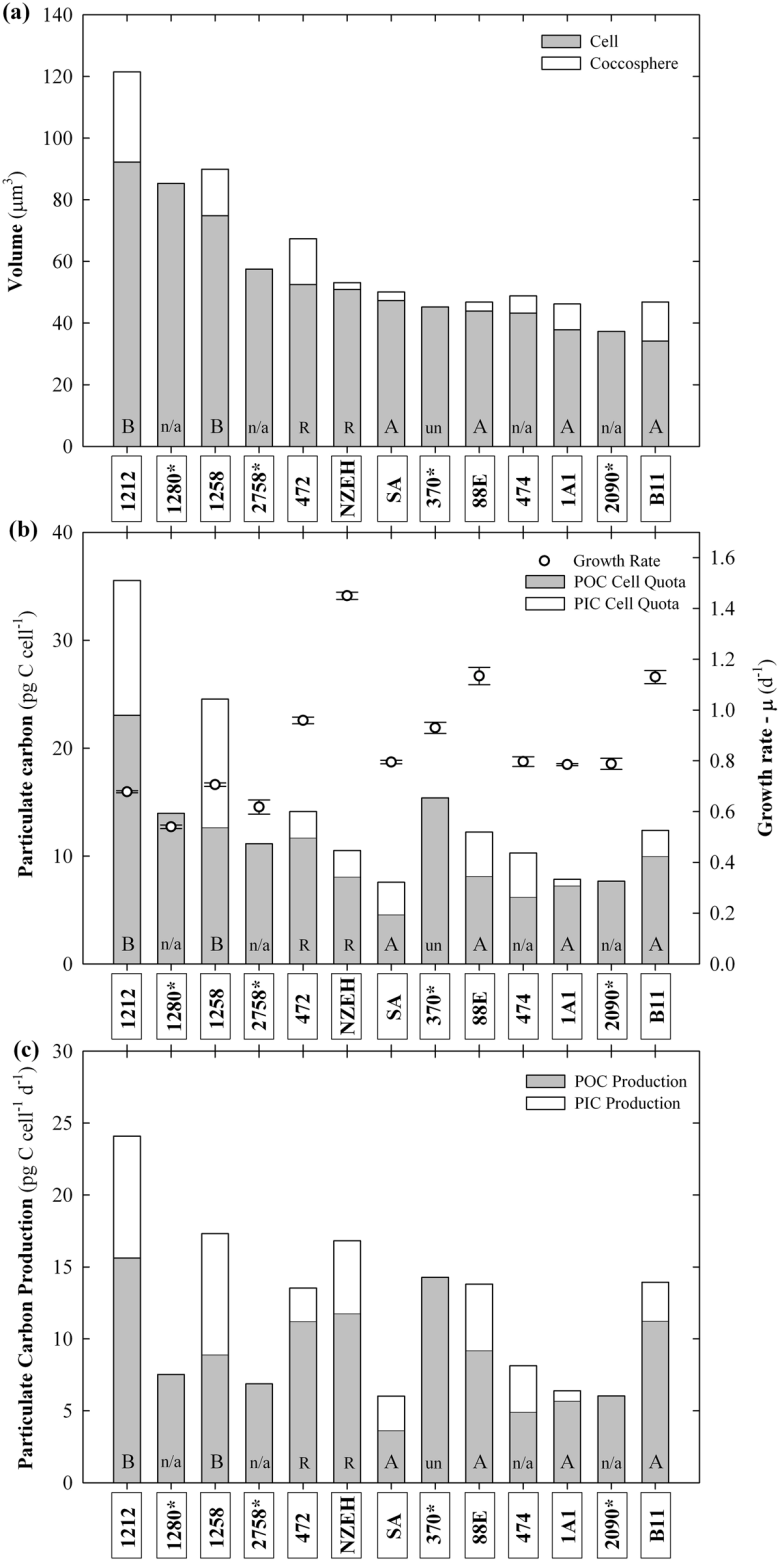
Histogram of three physiological parameters. (A) Cell size; cell volume (grey area) and coccolith layer (white area), which includes the attached coccoliths; (B) Growth rate (μ) and particulate organic and inorganic carbon cell quota; and (C) particulate organic and inorganic carbon production, comparing the13 strains grown under identical environmental conditions. (*) Indicates non-calcifying strains. Characters at the base of the bars indicate the morphotype corresponding to each strain. “n/a” indicates that there is not an available morphotype classification for that strain. Values were calculated as averages of three replicates. Error bars represent standard deviation between replicates.

Coccosphere (cell + coccoliths) volume was statistically different between strains (P <0.001) varying between 46.18 ±3.58 μm^3^ (M184CCMP1A1) and 121.45 ±25.82 μm^3^ (RCC1212) (Table 2). After coccoliths dissolution, the cell volume varied between 34.13 ±0.10 (B92/11) and 92.18 ±12.48 μm^3^ (RCC1212) (Fig 1). Inter-strain differences in cell volume were statistically different between strains (P <0.001).

**Table 2:** Physiological parameters measured in this study and collected from the literature.

Strain	μ (d^-1^)	Volume (μm^3^)		Cell Quota (pg cell^-1^)				Cell Ratios (pmol/pmol)		Reference
		Coccosphere	Cell	TPC	PIC	POC	PON	PIC:POC	C:N	
CCMP370*	0.93 ±0.02	-	45.23 ±2.63	15.38 ±2.12	-	11.52 ±1.02	1.74 ±0.15	-	10.29 ±0.62	This study
CCMP2758*	0.61 ±0.03	-	57.48 ±0.45	11.13 ±0.78	-	9.97 ±0.00	1.46 ±0.20	-	8.96 ±0.65	This study
B92/11	1.12 ±0.03	46.82 ±0.00	34.12 ±0.00	12.36 ±2.21	2.38 ±0.43	9.97 ±1.78	1.26 ±0.19	0.23 ±0.01	8.99 ±0.63	This study
M181CCMP88E	1.13 ±0.03	46.77 ±6.26	43.86 ±2.22	12.21 ±2.68	4.09 ±2.23	8.12 ±1.03	1.28 ±0.21	0.50 ±0.25	7.30 ±0.73	This study
AC474	0.79 ±0.02	48.81 ±6.48	43.20 ±0.00	10.27 ±1.46	4.06 ±1.99	6.20 ±0.69	0.63 ±0.22	0.65 ±0.40	12.37 ±2.69	This study
RCC1258	0.70 ±0.01	89.90 ±5.12	74.82 ±8.09	24.88 ±1.21	11.91 ±0.40	12.63 ±0.82	1.18 ±0.03	0.94 ±0.02	12.07 ±0.05	This study
M184CCMP1A1	0.78 ±0.01	46.18 ±3.58	37.78 ±0.93	7.85 ±2.16	0.59 ±0.64	7.25 ±1.54	1.16 ±0.14	0.12 ±0.05	5.48 ±0.73	This study
CCMP2090*	0.78 ±0.02	-	37.27 ±0.00	7.66 ±0.41	-	7.18 ±2.46	1.06 ±0.15	-	8.27 ±0.90	This study
CCMP1280*	0.53 ±0.01	-	85.28 ±0.00	15.64 ±1.45	-	13.94 ±2.10	1.51 ±0.14	-	10.76 ±0.84	This study
South Africa	0.79 ±0.01	50.08 ±4.93	47.30 ±0.00	7.56 ±1.52	3.00 ±1.69	4.56 ±0.28	0.74 ±0.03	0.66 ±0.40	7.22 ±0.68	This study
RCC1212	0.67 ±0.01	121.45 ±25.82	92.18 ±12.48	35.53 ±0.58	12.49 ±1.22	23.04 ±1.80	2.43 ±0.23	0.54 ±0.10	10.96 ±0.44	This study
NZEH	1.45 ±0.01	53.10 ±1.97	50.88 ±0.00	10.50 ±0.78	2.42 ±1.81	8.07 ±1.05	1.36 ±0.07	0.43 ±0.05	6.90 ±0.55	This study
AC472	0.95 ±0.01	67.34 ±4.71	52.48 ±0.00	14.11 ±3.66	2.42 ±1.89	11.69 ±1.95	1.28 ±0.17	0.20 ±0.14	10.60 ±0.39	This study
***Global average***	***0*.*86 ±0*.*25***	***63*.*38 ±26*.*06***	***53*.*99 ±18*.*65***	***13*.*81 ±7*.*98***	***4*.*82 ±4*.*31***	***10*.*74 ±4*.*85***	***1*.*31 ±0*.*45***	***0*.*48 ±0*.*26***	***9*.*40 ±1*.*90***	***This study***
RCC1238	1.65 ±0.02	-	-	-	7.99 ±0.41	10.99 ±0.82	-	0.72 ±0.09	-	[27]
CCMP374	1.34 ±0.01	-	-							[30]
AC481	0.13 ±0.02	61.58 ±0.00	-	-	9.231	12.462	-	0.74 ±0.16	-	[31]
RCC1212	0.98 ±0.01	-	-	-	9.35 ±0.51	11.41 ±1.12	-	0.81 ±0.04	-	[27]
NZEH	0.61 ±0.13	55.43 ±9.59	-	-	-	11.34 ±5.81	-	-	7.29 ±0.41	[32]
NZEH	1.35 ±0.00	-	-	-	5.76 ±0.24	7.08 ±0.48	-	0.80 ±0.03	8.60 ±0.00	[30]

(*) Non-calcifying strains

#### Growth rate (μ)

The growth rate of *E*. *huxleyi* strains was significantly different between strains (P <0.001). It ranged from 0.53 ±0.01 d^-1^ to 1.45 ±0.01 d^-1^ (Fig 1). The fastest growth rate was observed in NZEH and the lowest in the non-calcifying strain CCMP1280. Non-calcifying strains showed lower growth rates between 0.53 ±0.01 d^-1^ and 0.93 ±0.02 d^-1^, whilst those of calcifying strains varied between 0.67 ±0.01 and 1.45 ±0.01 d^-1^ (Table 2). Growth rate of non-calcifying strains was on average 19.28% lower than in calcifying strains (P = 0.015). Growth rate was inversely correlated with coccosphere volume, cell volume and PIC cell quota (r = -0.522, -0.479 and -0.481, respectively; P <0.05) (S2 Table).

#### Particulate carbon

**Fig 2:**
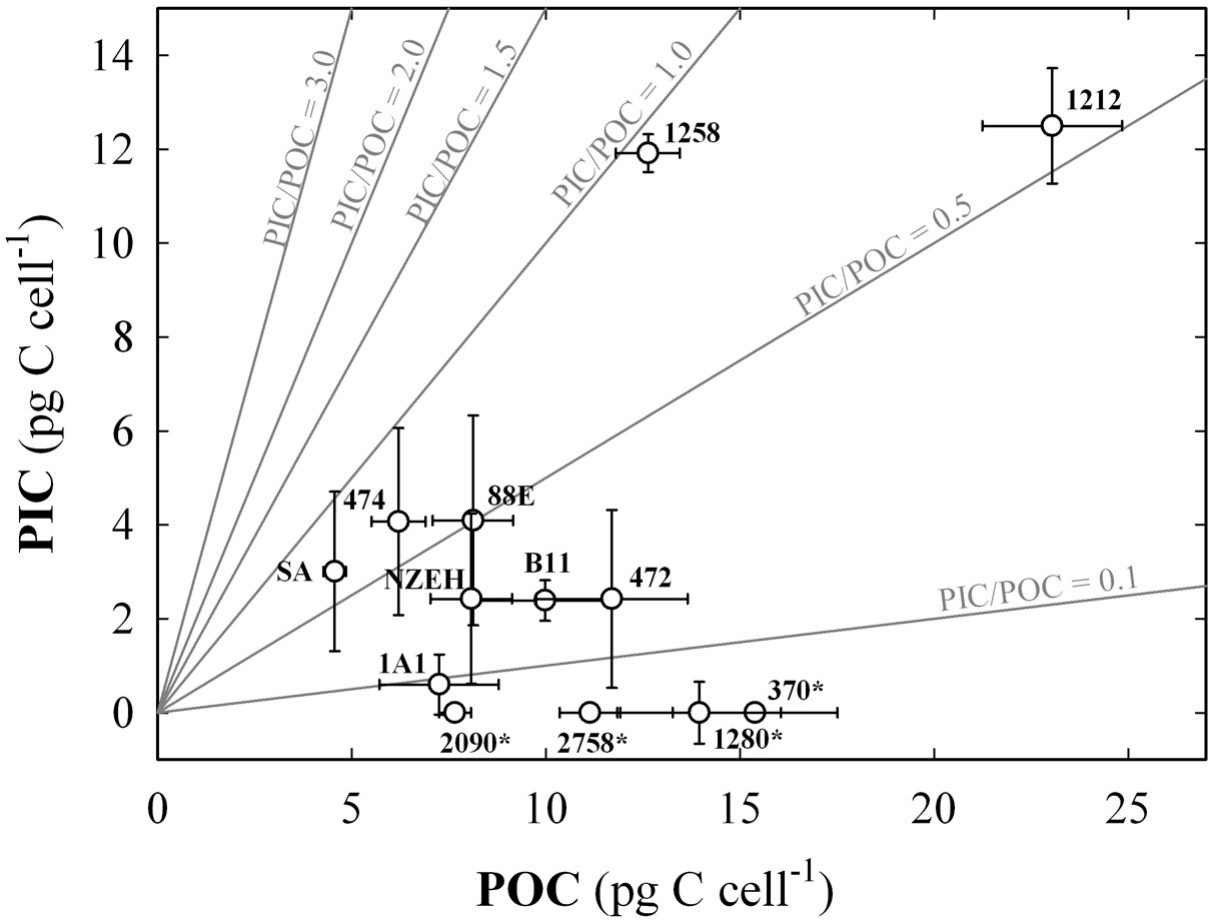
Particulate organic and inorganic carbon cell quota proportions for the 13 strains. Each value includes three replicates while the error bars represent the standard deviation. The numbers next to the symbols indicate the strain code. (*) indicates non-calcifying strains. Grey lines indicate the different particulate inorganic to organic carbon ratios (PIC:POC).

**Fig 3:**
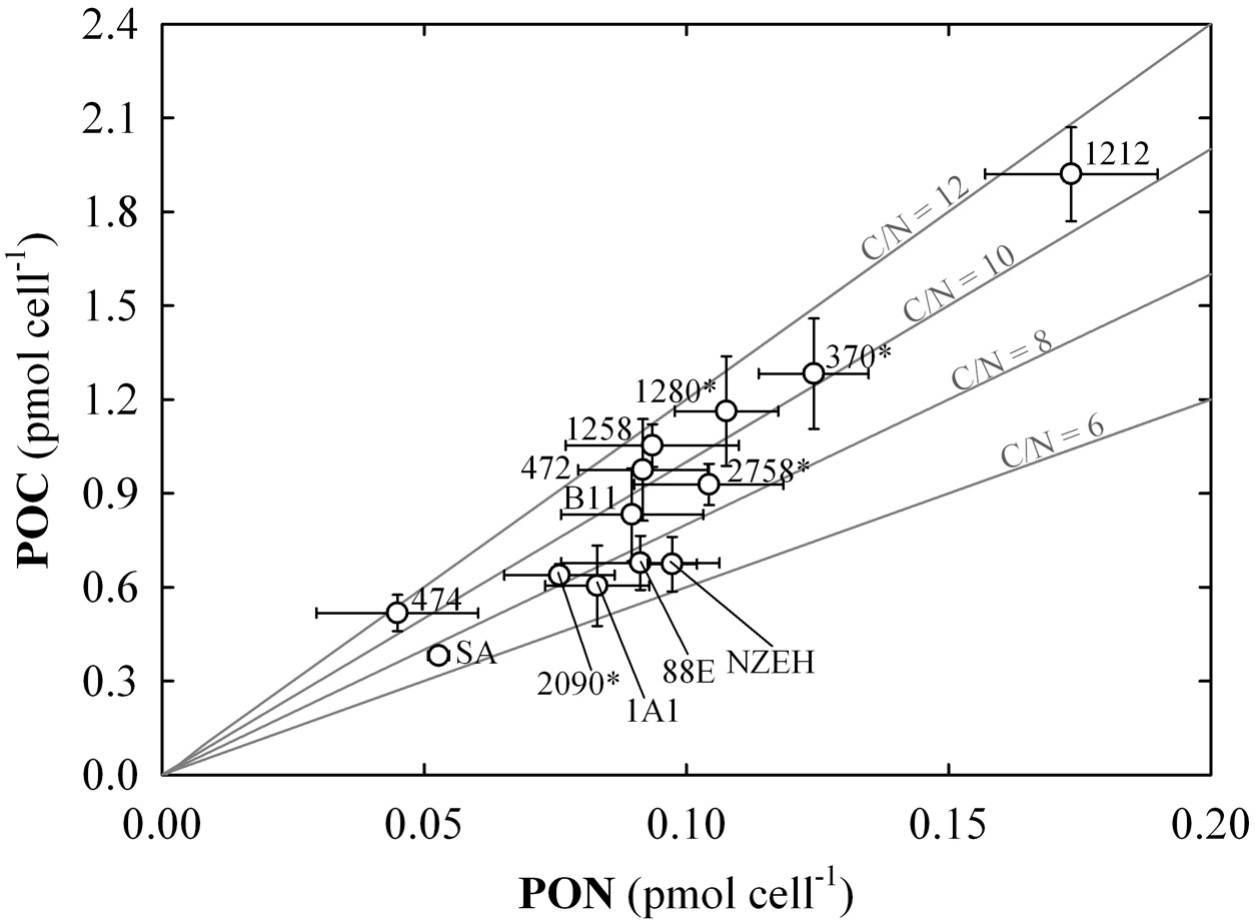
Particulate carbon to particulate nitrogen ratio (C:N) cell quotas. Each value includes three replicates (error bars represent the standard deviation). The numbers next to the symbols indicate the strain code. (*) indicates non-calcifying strains. Continuous grey lines indicate the different C:N.

POC cell quota ranged from 4.56 ±0.28 to 23.04 ±1.80 pg C cell^-1^ in the strains South Africa and RCC1212, respectively (Fig 1). Amongst the calcifying strains, PIC cell quota ranged from 0.59 ±0.64 to 12.49 ±1.54 pg C cell^-1^ in strains M184CCMP1A1 and RCC1212, respectively. These two parameters were directly correlated (r = 0.727; P <0.05). PIC cell quotas showed a direct correlation with coccosphere size and cell volume (*r* = 0.788 and 0.847, respectively; *P* <0.05). Similarly, POC cell quota was positively correlated with coccosphere volume and cell volume (*r* = 0.691 and 0.725; *P* < 0.05). Production of POC ranged from 3.62 ±0.22 pg C cell^-1^ d^-1^ in South Africa strain to 15.62 ±1.25 in RCC1212, and PIC production ranged from 0.69 ±0.45 pg C cell^-1^ d^-1^ in M184CCMP1A1to 8.46 ±0.81 pg C cell^-1^ d^-1^ in RCC1212 (Fig 1). The average PIC:POC ratio for the calcifying strains was 0.48 ±0.26 (Fig 2). The highest values of PIC:POC were observed in RCC1258 (0.94 ±0.02) and South Africa (0.66 ±0.40) strains (Fig 3). All the particulate carbon parameters tested for the *E*. *huxleyi* were statistically different between strains (P <0.001), with exception of PIC/POC were P = 0.127 (S2 Table).

#### Particulate Nitrogen

Average PON was 1.31 ±0.45 pg N cell^-1^ for the 13 strains used in this study. The range of variation was between 0.63 ±0.22 and 2.43 ±0.23 pg N cell^-1^. The PON showed a direct significant correlation with POC and PIC cell quota, and cell volume (r = 0.917; 0.559 and 0.583, respectively; P <0.05). The carbon to nitrogen ratio (C:N) ranged between 5.48 ±0.73 to 12.37 ±2.69, observed in NZEH and AC474, respectively. Average C:N for all the *E*. *huxleyi* strains was 9.40 ±1.90 pmol:pmol (Fig 3). The C:N ratio was inversely correlated with growth rate (r = -0.451; P <0.05). Both, PON and C:N were statistically different between strains (P <0.001).

### Biogeochemical variation in calcite composition

**Fig 4:**
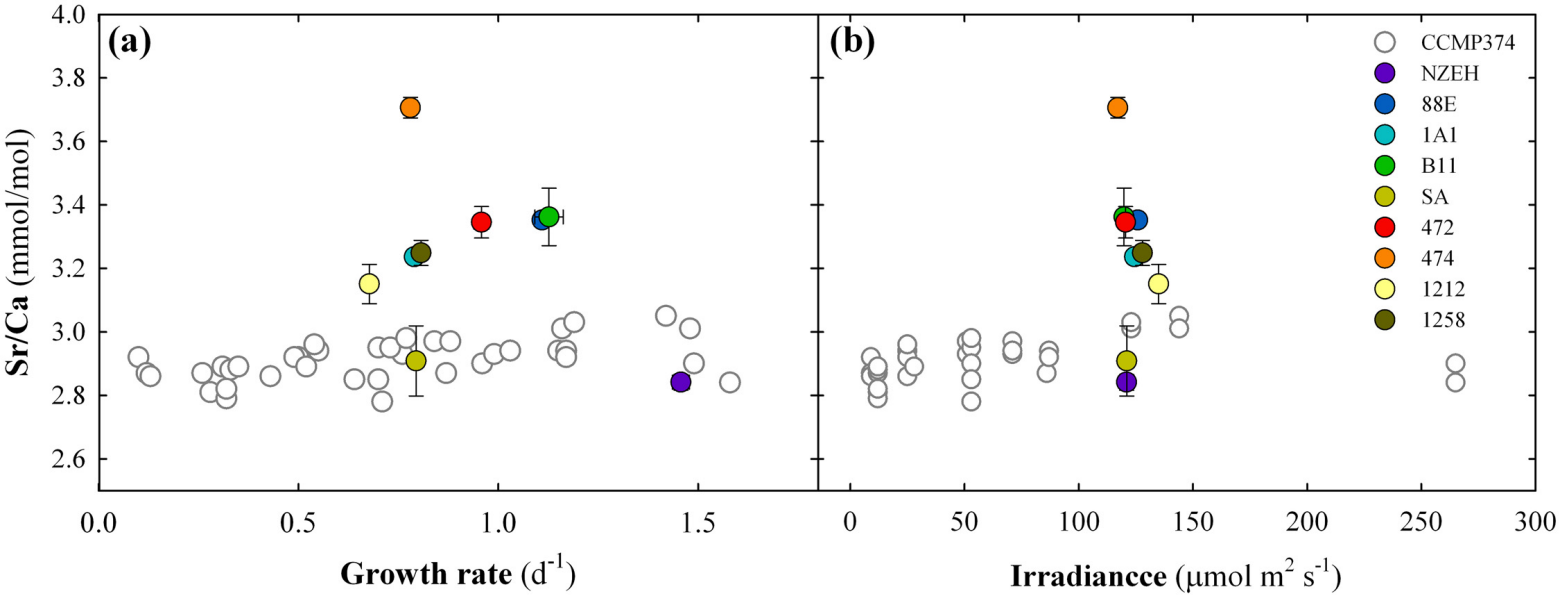
Comparison of the strontium to calcium ratios (Sr:Ca) from the 9 calcifying strains with the Sr:Ca from a single strain cultured under different irradiance levels. White symbols represent single values compiled from Stoll *et al*. [42]. Colored symbols represent mean values of three replicates obtained from our laboratory cultures. Error bars are standard deviation of three replicates.

Calcite Sr:Ca ratio was measured in triplicates in the 9 calcifying strains of *E*. *huxleyi* (NZEH, RCC1212, South Africa, AC474, AC472, RCC1258, B92/11, M184CCMP1A1 and M181CCMP88E). Average Sr:Ca was 3.28 ± 0.32 mmol:mol (Table 3) and the individual samples ranged from 2.75 ± 0.06 to 3.71 ± 0.03 mmol:mol (Fig 4). The strontium-partitioning coefficient (D_Sr_) ranged from 0.350 ± 0.007 to 0.475 ± 0.004. Both, Sr:Ca and D_Sr_ were statistically different between strains (P <0.001 and P = 0.034, respectively). The average calcite Mg:Ca was 0.39 ± 0.06 mmol:mol (Table 2). The indicators of organic phase’s contamination (phosphorus (P):Ca and iron (Fe):Ca) [37] were below 6.5 and 41 mmol:mol, respectively.

**Table 3:** Magnesium (Mg) and strontium (Sr) concentrations in culture medium and in coccolithophore calcite, and the corresponding partitioning coefficients (Dr_x_).

Strain	n^a^	Ca test^b^	Seawater		Calcite (mmol/mol)		Contamination proxy (mmol/mol)		Partitioning coeficient	
			Mg:Ca^c^	Sr:Ca^d^	Mg:Ca^a^	Sr:Ca^e^	P:Ca^a^	Fe:Ca^a^	D_Mg_^a^	D_Sr_^e^
NZEH	2	19 ± 1	5.52	7.66	0.15 ± 0.01	2.86 ± 0.03	0.80 ± 0.23	8.34 ± 5.64	0.027 ± 0.003	0.374 ± 0.004
RCC1212	2	21 ± 2	5.59	7.71	0.45 ± 0.11	3.17 ± 0.06	1.28 ± 0.14	40.53 ± 6.54	0.081 ± 0.027	0.394 ± 0.007
South Africa	1	17 ± 0	5.60	7.74	0.55 ± 0.00	2.75 ± 0.06	6.10 ± 1.16	30.37 ± 4.03	0.099 ± 0.000	0.350 ± 0.007
AC474	3	23 ± 21	5.62	7.81	11.76 ± 6.25	3.71 ± 0.03	250.56 ± 164.49	723.37 ± 474.17	2.091 ± 1.298	0.475 ± 0.004
AC472	2	115 ± 18	5.63	7.87	5.46 ± 0.64	3.37 ± 0.06	114.30 ± 14.15	190.88 ± 16.13	0.970 ± 0.149	0.428 ± 0.008
RCC1258	3	130 ± 25	5.58	7.75	6.17 ± 0.30	3.26 ± 0.03	40.70 ± 11.40	221.34 ± 75.75	1.102 ± 0.061	0.421 ± 0.004
B92/11	2	16 ± 1	5.59	7.68	8.01 ± 2.01	3.29 ± 0.23	266.40 ± 60.70	755.41 ± 165.53	1.434 ± 0.475	0.428 ± 0.029
M184CCMP1A1	3	14 ± 9	5.62	7.74	13.11 ± 4.38	3.56 ± 0.40	261.15 ± 141.57	836.15 ± 430.74	2.333 ± 0.867	0.460 ± 0.051
M181CCMP88E	3	6 ± 6	5.59	7.71	17.57 ± 3.99	3.55 ± 0.54	240.69 ± 122.57	1107.04 ± 141.92	3.143 ± 0.452	0.460 ± 0.070
**Average**			**5.59 ±0.03**	**7.74 ±0.07**	**0.39** ^§^**± 0.06**	**3.28 ± 0.32**	**-**	**-**	**0.069** ^§^**±** 0.037	**0.421** ^§^**± 0.042**

^(a)^ number the of samples considered after removal of organic phases for Mg/Ca, P/Ca, Fe/Ca and D_Mg_;

^(b)^ Indicates the concentration of calcium in the sample (ppm) after removal of organic phases;

^(c)^ mol/mol;

^(d)^ mmol/mol;

^(e)^ values calculated from 3 replicates;

^(§)^ Mean value of the strains NZEH, RCC1212 and South Africa, accepted as effectively clean of organic phases.

## Discussion

### Experimental set-up considerations

Many experiments have investigated the physiological response of *E*. *huxleyi* under future ocean acidification scenarios obtaining contrasting results (summary in [9]) but recently a unifying concept was developed [43], showing that the shifting in optimal *p*CO_2_ observed in different strains is also affected by culture conditions such as light and temperature [43]. Intra-species variability has been suggested as key factor for the recorded variability in physiological responses [9, 27, 44]. However, the large variety of experimental set ups, environmental conditions and different investigated strains hamper any attempt to estimate the magnitude of this phenotypic variability. Our approach suggests that phenotypic variability of physiological and biogeochemical traits between strains grown under identical environmental conditions may reflect genotypic differences. Thus, we examined phenotypic variability amongst 13 strains representative of different ocean biogeographic provinces grown under identical culture conditions.

We acknowledge that the chosen environmental conditions do not reflect strain specific optimal conditions neither do they represent the biogeographic conditions at the site of isolation. Some of the investigated strains have been maintained in culture collections for decades. For example, strain “South Africa” (Table 1) has been kept in culture for 30 years. In these cases, the genetic drift and adaptation to culture conditions over time represents a potential bias given that the phenotype of the strains investigated may no longer accurately represent the initially isolated cells. In general, older cultures are more likely to have departed from their “natural state” than newer isolates, especially in terms of traits that are not under selection under culture conditions [45]. Yet, most of the strains were widely used in many research studies during the last 20 years (Table 1). Therefore, our results (S2 Table) are relevant for comparison of literature strain-specific responses.

### Biogeographic considerations

Despite several studies showing a genetic separation between strains originating in the northern and southern hemisphere [12, 14, 13], physiological and biogeochemical parameters measured in our experiments do not separate between strains from the two hemispheres. For instance, the two B morphotypes included in this study, strain 1212 (from southern hemisphere) and 1258 (from northern hemisphere), showed similar biogeochemical properties (S2 Table). The morphometric analysis performed here suggests that A type strains have typically small volumes and that B type strains possess the largest cell volumes (Fig 1). However, the small number of strains used in this study might prevent us from drawing conclusive results in relation to the biogeographic origin of the strains.

Latitudinal origin of isolation was not reflected in the variation of physiological parameters or morphotype identities. Thus, at ~19°C we would expect to find strains from mid-latitudes growing at optimum conditions, while the same temperature for strains isolated at high latitudes might be the upper or lower threshold for growth depending on the average annual temperature at the site of isolation [46, 47]. However, other physico-chemical parameters such as light regimes and seawater CO_2_ concentrations could shift the maximum peaks of the physiological and biogeochemical properties under the same temperature [25, 43, 48]. Our results are in line with those obtained by Reid *et al*. [49], where functional diversity based on enzyme activity assays of 52 strains of *E*. *huxleyi* showed no significant effects of strains grouped according to biogeographic origin.

### Strain-specific significance in biogeochemical cycling of elements

A mechanistic relationship between particulate organic and inorganic carbon, and growth rates was suggested by Buitenhuis *et al*. [50]. However, we only find a moderate inverse correlation between growth rate and cell volume (r = -0.479; P = 0.002), POC production (r = -0.59; P <0.001) and C:N (r = -0.441; P = 0.005). Our results suggest that strains with higher cell volume entail lower growth rate and higher carbon production (r >0.73; P <0.001) (S2 Table). Therefore, the PIC and POC production are strongly determined by cell volume, and hence PIC and POC cell quota. The variation of PIC and POC production, and the PIC:POC ratios are in agreement with values previously reported in the literature [27, 32].

It is surprising that the range of strain-specific phenotypic variability for the different parameters determined here is much higher than the maximum variation registered in previous studies for a single strain growing under increasing concentrations of seawater CO_2_ [27]. For instance, the maximum variation of growth rate, POC and PIC cell quota, within a range of about 900 μatm of CO_2_, determined by Langer *et al*. [27], corresponds to only 49, 25, and 23%, respectively, of the variation determined in the present study for 9 strains under identical environmental conditions. This suggests that the inter-strain genetic variability has greater potential to induce larger phenotypic differences than the phenotypic plasticity of a single strain cultured under a broad range of variable environmental conditions. In the ocean, the shifts in PIC:POC ratios in a population are likely to be more strongly controlled by the changes in population composition (dominating strains) than the plasticity of PIC:POC of a given strain as suggested by the results obtained by Beaufort *et al*. [51]. Thus, the combination of strain-specific values of organic and inorganic carbon cell quotas, and growth rates represent the strain-specific biogeochemical signature with potential to specifically contribute to the biological carbon pump when it dominates large blooms (Fig 1).

The range of C:N variability determined in this study (between 5.48 and 12.37) covers the systematic variation between phytoplankton groups reported in Quigg *et al*. [52]. Interestingly, the direct correlation between C:N ratios and cell volume (r = 0.47, P<0.005), along with the inverse correlation with growth rate (r = -0.44, P = 0.005), reveals hints on the implication of cell size on biogeochemical cycling of elements [53]. Thus, the nitrogen remineralization rate in the water column could be largely affected by cell volume, which is strain-specific. The strain-specific stoichiometric variations have implications on the export of carbon and other elements (N, P, etc.) to the deep ocean.

Coccolithophores are commonly integrated in biogeochemical models because they dominate pelagic PIC production and export. Models largely rely on the limited experimental datasets available for PIC production. Extrapolations from experimental work on single strains might lead to over/underestimated results when not taking into account the genetic diversity and ecophysiological plasticity within the species [43, 48]. For instance, if we compare the range variation of PIC:POC ratios recorded in our study (between 0.12±0.05 and 0.94±0.02) with the parameterization used by Gehlen *et al*. [54] this issue becomes evident. The range of PIC:POC variation registered in our study with saturation state with respect to calcite (Ω_Calcite_) = 3.75±0.25 was 1.2-fold larger than that recorded for a single strain of *E*. *huxleyi* (0.3–1.0 [55]) within range from 2 to 10 Ω_Calcite_, and for a mesocosm bloom dominated by the same species (0.3–0.9 [56]). Therefore, finding a way to include *E*. *huxleyi*'s functional diversity in biogeochemical models is essential to improve our understanding about the role of this species on global biogeochemical cycling of elements and the effect of climate change-driven selection on the populations. However, the lack of systematic biogeographic information on physiological parameters complicates the integration into models.

### Strain-specific elemental chemistry

The calcite Sr:Ca ratio for each strain was well within the range of reported values from other culture experiments [42, 57]. No correlation was found between calcite Sr:Ca ratios, growth rates, cell and coccosphere volume, and calcification rates (*P* > 0.05). However, it should be noted that six strains (excluding the three outermost calcite Sr:Ca of strains AC474, SA and NZEH) follow a positive trend of increasing calcite Sr:Ca along with increasing growth rate, as previously described by Stoll *et al*. [58]. The range of variation of calcite Sr:Ca for the 9 calcifying strains used for elemental analyses (from 2.75 to 3.57 mmol:mol) was 3 times higher than the Sr:Ca range (2.78 to 3.05 mmol:mol) detected in a single strain of *E*. *huxleyi* (CCMP374) cultured at different light levels [42] (Fig 4). The strains displaying the smallest and largest ratios (SA and AC474, respectively) were not isolated at high latitudes, therefore, we can discard the effect of adaptation to a baseline temperature lower than the 19.3°C used in this study. Since all the strains were cultured under nutrient replete conditions, and harvested during the exponential growth phase, physiological control is assumed to be similar for all strains. Differences in the calcite Sr:Ca ratio may be due to inherent genomic properties controlling seawater Sr transport mechanisms, both, at the level of extracellular membrane and the coccolith vesicle, where calcification takes place [59]. Thus the incorporation of Sr into the calcite lattice of *E*. *huxleyi* is strain-specific.

The wide calcite Mg:Ca ratio variation was influenced by incomplete removal of organic matter that can bias intra-species comparisons (Table 2) when calcite content is low in relation to POC, i.e., in low calcifying strains [37]. Given that the organic phases of phytoplankton contain an order of magnitude more Mg (~560 mmol:mol of P) than, for example, Sr (~54 mmol:mol of P) [60], removal of organic phases is critical in determining Mg in *E*. *huxleyi* [43] owing to the small size of the samples and the low calcite content (~40 ppm Ca; see Table 2). In our study, only the highest calcifying strains displayed relatively low organic matter content as indicated by the P:Ca and Fe:Ca ratios, which were used as indicators of organic contamination in the calcite samples [37] (Table 2). After the cleaning pre-treatment only 5 samples from 3 strains (SA, 1212 and NZEH) satisfied the requirements established to consider the measurement of calcite Mg:Ca ratio as acceptable: (1) Fe:Ca ratio < 50 mmol:mol, and (2) P:Ca < 6 mmol:mol [37]. We conclude that Mg/Ca ratios cannot be used with confidence for *E*. *huxleyi* due to the difficulties to remove organic phases from the samples. We suggest that *E*. *huxleyi* is not a good candidate for measuring calcite Mg:Ca, and thus should not be used to reconstruct any property using the geological record. However, in larger species such as *Gephyrocapsa oceanica*, *Coccolithus pelagicus*, or *Calcidiscus leptoporus* the Mg organic phases are easier to clean, and thus calcite Mg:Ca measurements are more reliable for applications in paleo-reconstructions [25, 37].

We show a relationship between intra-species diversity and variability in coccolith geochemistry, which is an important factor in proxy calibration of primary productivity (Sr:Ca) [8, 24, 25]. In addition, organic biomarker proxies (alkenone-derived carbon isotopes fractionation) to determine ancient *p*CO_2_ scenarios [61] are affected by strain-specific variability on cell volumes and POC quotas [62]. In order to obtain an accurate calibration of productivity, temperature, and *p*CO_2_, strain biogeochemical identity within the species concept of *E*. *huxleyi* needs to be considered. Therefore, inter-strain variability will have to be incorporated into the palaeopoxy calibration and its error margins.

Inter-strain complexity driven by strain functional diversity has the potential to mask variations in elemental composition associated to interactions with environmental conditions due to strain-specific acclimation patterns and element discrimination capacity [63]. This complexity might limit the direct use of elemental ratios as paleoproxies. However, changes on calcite Sr:Ca could be used to detect and characterize climate driven changes in dominant strains, which could shed light on determining what and how climatic events drive changes in coccolithophore community composition and evolution [53].

### Implications for climate change and ocean acidification research

The impact of climate change on coccolithophores predicted for the next 100 years as a result of anthropogenic activity will ultimately depend on their capability to adapt. The frequency of environmental changes will determine adaptation; i.e., new beneficial mutations, horizontal gene transfer, or recombination [64, 65]. The genetic variability and different metabolic repertoires of *E*. *huxleyi* have the potential to make this species particularly resilient in novel environments [11]. Additionally, the phenotypic variability of *E*. *huxleyi* showed in this study constitutes an enormous advantage to mitigate the effects of rapid climate change, since physiological functioning of phytoplankton is expected to be phenotypically buffered against such environmental heterogeneity [66]. Thus, high diversity of environmentally induced responses [27, 67] has the potential to lead to different evolutionary outcomes [68]. As a counterpoint, the complexity in intra-species resilience, adaptation and competition of *E*. *huxleyi* might complicate the application of paleoproxies to reconstruct ancient climate scenarios, predictions of the ocean carbon pump strength and efficiency, the biogeochemical cycles and ecological impacts in future climate scenarios.

## Conclusion

Phytoplankton intra-species genetic variability is well studied [69, 70]. Yet, this study provides additional evidence of intra-species physiological variability of the coccolithophore species *E*. *huxleyi*, revealing strain-specific elemental signatures and growth rates. Thus, it can be expected that blooms dominated by a certain strain have different signatures in the biological pump and the elements they export. Most of the strains included in this study have previously been used in laboratory experiments investigating the response of coccolithophores to projected global change (increase in seawater CO_2_, temperature, nutrients) showing variable responses [9]. Our results provide a frame of comparison for the phenotypes that have been used in many laboratory and model studies. In addition, the great variability reflected in the calcite elemental composition and the physiological parameters highlight the need to take in account the intra-species for the calibration of paleo-proxies.

## Supporting Information

S1 Fig Comparison of carbon measurements. We used particulate carbon parameters (PIC, POC and TPC) determined with two different techniques: (1) carbon concentration using an elemental analyzer, and (2) calcium concentration using ICP-AES, and results were compared. This was done to compare the two most used carbon-measuring techniques in the literature and assess possible errors or deviations.
(DOC)

S1 Notes Comparison of particulate carbon parameters (PIC, POC and TPC) determined using two different techniques. (DOC)

S1 Table Culture medium’s carbonate system of our strains and of published strains. (DOC)

S2 Table Pearson product-moment correlation coefficients between all parameters measured on the 13 different *Emiliania huxleyi* strains cultured under identical environmental conditions. (DOC)
