# Supplementary material for: Phenotypic Variability in the Coccolithophore Emiliania huxleyi
Source: PLoS One. 2016 Jun 27;11(6):e0157697. doi: 10.1371/journal.pone.0157697 (PMC4922559; doi:10.1371/journal.pone.0157697)

**Supporting information**

**S1 Fig. Comparison of carbon measurements.** We used particulate carbon parameters (PIC, POC and TPC) determined with two different techniques: (1) carbon concentration using an elemental analyzer, and (2) calcium concentration using ICP-AES, and results were compared. This was done to compare the two most used carbon-measuring techniques in the literature and assess possible errors or deviations.


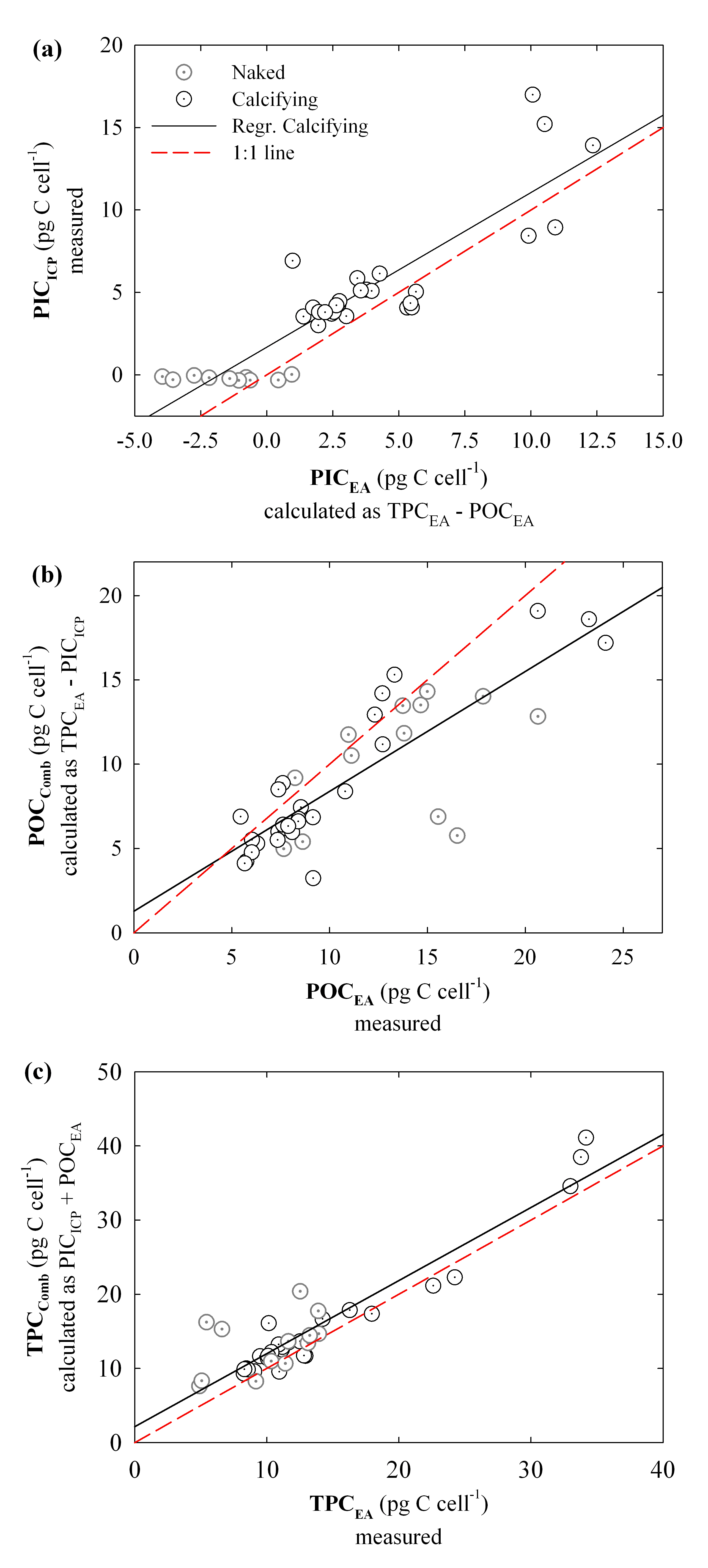

Supplement: S1 Fig — We used particulate carbon parameters (PIC, POC and TPC) determined with two different techniques: (1) carbon concentration using an elemental analyzer, and (2) calcium concentration using ICP-AES, and results were compared. This was done to compare the two most used carbon-measuring techniques in the literature and assess possible errors or deviations. (DOC) [file pone.0157697.s001.doc]
