# Supplementary material for: Phenotypic Variability in the Coccolithophore Emiliania huxleyi
Source: PLoS One. 2016 Jun 27;11(6):e0157697. doi: 10.1371/journal.pone.0157697 (PMC4922559; doi:10.1371/journal.pone.0157697)
Supplement: S1 Notes — (DOC) [file pone.0157697.s002.doc]

**Supporting information**

**S1 Notes.** Particulate carbon parameters (PIC, POC and TPC) were determined using two different techniques: (1) carbon concentration using an elemental analyzer, and (2) calcium concentration using ICP-AES, and results were compared. When PIC_EA_ vs PIC_ICP_ were correlated, the measurements of calcifying strains slightly derived from the 1:1 line, especially at lower concentrations of PIC. PIC was derived from ICP measurements of non-calcifying strains (which are expected to have no calcite, thus PIC should be 0) and ranged from 0.01 and -0.35 (a 0.36 pg C units variation). However, PIC derived from AE measurements of exactly the same samples showed a wider range of variation between 0.95 and -11.09 (12.04 pg C units variation) (Figure 1A). When POC_EA_ and POC_Comb_ (calculated as TPC_EA_ - PIC_ICP_) were compared, the shift from the 1:1 line was higher (Figure 1B). However, the values of TPC_Comb_ derived from combination of EA and ICP (PIC_ICP_ + POC_EA_), are very similar to TPC_EA_, with less data dispersion and similar slope to the 1:1 line (Fig 1C).

**The comparison of the particulate carbon results obtained using EA or ICP, or a combination of both shows that there is a shift of ~ 2 pg C units in the final dataset depending on the technique used (although in some samples the shift was as high as ~ 7 pg C). On one hand, PIC determination using EA requires an intermediate step of acidification (POC samples are fumed with H_2_SO_4_ in order to dissolve inorganic carbon in the samples), both TPC and POC samples need to be packed with aluminum tin in small pellets, and finally PIC is calculated by subtracting the two independent samples (TPC - POC). On the other hand, PIC determination using ICP equipment only requires dissolution of the sample with HNO_3_ and accurate determination of the acid volume added. Inorganic carbon concentration is calculated following Ca concentrations based on the assumption that all the Ca measured in the sample is CaCO_3_ and the concentration of Ca in organic phases is negligible (for non-calcifying phytoplankton average Ca/C is 0.0034 mol/mol (Ho et la 2003). The simplicity of the second method, the fact that all the intermediate manipulations required to prepare the sample for EA analysis represent a potential source of error associated with the manipulation of the sample and a potential source of contamination or sample loss, and that the detection limit of the ICP method is much lower and shows higher precision (S1 Fig), means that the ICP technique is more suitable to determine PIC. However, the ICP method does not allow to determine organic carbon or nitrogen, therefore, it relies on the EA method to determine one of the parameters, POC/PON or TPC/TPN, which are required to quantify and characterize the particulate carbon/nitrogen in the sample. Our results indicate that when using POC_EA_ and TPC_EA_ in combination with PIC_ICP_, the POC_EA_ data provide the most robust results (S1 Fig).**
