# Supplementary material for: Phenotypic Variability in the Coccolithophore Emiliania huxleyi
Source: PLoS One. 2016 Jun 27;11(6):e0157697. doi: 10.1371/journal.pone.0157697 (PMC4922559; doi:10.1371/journal.pone.0157697)
Supplement: S1 Table — (DOC) [file pone.0157697.s003.doc]

**Supporting information**

**S1 Table. Culture medium’s carbonate system of our strains and of published strains.**

| **Strain** | **TA** | **DIC** | **pH_total_** | ***p*CO_2_** | **HCO_3_^-^** | **CO_3_^2-^** | **Ω Calcite** | **Reference** |
| --- | --- | --- | --- | --- | --- | --- | --- | --- |
|  | (µmol kg-1 SW) | (µmol kg-1 SW) |  | *(µatm)* | (µmol kg-1 SW) | (µmol kg-1 SW) |  |  |
| CCMP370* | 2284.27 ±4.00 | 2037.34 ±3.01 | 8.02 ±0.05 | 413.50 ±2.77 | 1847.96 ±3.58 | 175.80 ±0.70 | 4.20 ±0.02 | This study |
| CCMP2758* | 2288.60 ±4.36 | 2048.95 ±4.10 | 8.01 ±0.01 | 425.08 ±12.37 | 1863.12 ±7.59 | 171.85 ±4.41 | 4.10 ±0.11 | This study |
| B92/11 | 2283.04 ±24.36 | 2065.16 ±3.75 | 7.96 ±0.04 | 479.40 ±42.87 | 1892.27 ±8.35 | 156.86 ±13.38 | 3.70 ±0.31 | This study |
| M181CCMP88E | 2279.01 ±29.50 | 2065.46 ±13.03 | 7.95 ±0.01 | 497.55 ±41.73 | 1894.24 ±9.16 | 154.95 ±12.47 | 3.68 ±0.30 | This study |
| AC474 | 2280.74 ±3.73 | 2067.18 ±4.21 | 7.97 ±0.02 | 492.30 ±7.29 | 1895.71 ±4.97 | 155.40 ±1.65 | 3.71 ±0.04 | This study |
| RCC1258 | 2246.86 ±29.34 | 2042.05 ±27.66 | 7.93 ±0.02 | 501.44 ±14.60 | 1876.64 ±25.80 | 149.01 ±3.30 | 3.56 ±0.08 | This study |
| M184CCMP1A1 | 2266.52 ±2.24 | 2061.59 ±9.40 | 7.93 ±0.02 | 525.14 ±32.73 | 1895.64 ±14.20 | 148.87 ±5.89 | 3.52 ±0.14 | This study |
| CCMP2090* | 2295.35 ±3.16 | 2081.97 ±12.78 | 8.03 ±0.00 | 493.93 ±33.27 | 1909.85 ±19.13 | 155.91 ±7.61 | 3.73 ±0.18 | This study |
| CCMP1280* | 2286.66 ±2.87 | 2045.05 ±28.58 | 8.02 ±0.05 | 414.52 ±60.58 | 1858.26 ±43.06 | 172.97 ±16.57 | 4.13 ±0.40 | This study |
| South Africa | 2266.49 ±0.64 | 2060.16 ±13.71 | 7.95 ±0.03 | 498.72 ±37.67 | 1893.19 ±21.00 | 150.50 ±8.61 | 3.59 ±0.21 | This study |
| RCC1212 | 2235.51 ±4.00 | 2037.75 ±9.84 | 7.96 ±0.01 | 520.56 ±28.78 | 1876.34 ±14.32 | 144.45 ±5.72 | 3.45 ±0.14 | This study |
| NZEH | 2251.3 ±14.88 | 2051.99 ±9.84 | 7.95 ±0.01 | 499.92 ±9.82 | 1889.88 ±7.01 | 145.27 ±3.70 | 3.46 ±0.09 | This study |
| AC472 | 2276.81 ±3.36 | 2055.38 ±12.94 | 7.96 ±0.02 | 475.52 ±23.63 | 1879.72 ±17.98 | 160.26 ±5.86 | 3.82 ±0.14 | This study |
| ***Average*** | ***2272.40 ±18.04*** | ***2055.39 ±13.16*** | ***7.97 ±0.04*** | ***479.82 ±37.96*** | ***1882.52 ±17.64*** | ***157.08 ±10.47*** | ***3.74 ±0.25*** | ***This study*** |
|  |  |  |  |  |  |  |  |  |
| RCC1238 | 2243.01 ±83.44 | 2044.50 ±7.78 | 7.95 ±0.16 | 534.04 ±199.89 | 1882.50 ±34.65 | 144.50 ±48.79 | 3.55 ±1.20 | [28] |
| CCMP374 | 2260.01 ±10.00 | 1980.00 ±50.00 | 8.08 ±0.02 | 345.19 ±0.00 | 1772.34 ±0.00 | 196.51 ±0.00 | 4.71 ±0.00 | [32] |
| AC481 | 2057.11 ±17.11 | 1863.25 ±28.07 | 7.95 ±0.07 | 464.10 ±37.48 | 1863.25 ±28.07 | 139.80 ±5.94 | 3.31 ±0.14 | [33] |
| RCC1212 | 2313.01 ±0.00 | 2066.00 ±0.00 | 8.05 ±0.00 | 407.00 ±0.00 | 1877.00 ±0.00 | 176.00 ±0.00 | 4.30 ±0.00 | [28] |
| NZEH | 2192.12 ±33.52 | 1975.33 ±6.10 | 7.97 ±0.07 | 469.28 ±88.30 | 1799.24 ±29.01 | 160.37 ±25.90 | 3.85 ±0.62 | [31] |
| NZEH | 2260.01 ±10.00 | 1980.00 ±50.00 | 8.08 ±0.02 | 345.19 ±0.00 | 1772.34 ±0.00 | 196.51 ±0.00 | 4.71 ±0.00 | [32] |
