# Supplementary material for: Phenotypic Variability in the Coccolithophore Emiliania huxleyi
Source: PLoS One. 2016 Jun 27;11(6):e0157697. doi: 10.1371/journal.pone.0157697 (PMC4922559; doi:10.1371/journal.pone.0157697)
Supplement: S2 Table — (DOC) [file pone.0157697.s004.doc]

**Supporting information**

**S2 Table. Pearson product-moment correlation coefficients between all parameters measured on the 13 different *Emiliania huxleyi* strains cultured under identical environmental conditions.**

|  |  |  |  |  |  |  |  |  |  |  |  |  |
| --- | --- | --- | --- | --- | --- | --- | --- | --- | --- | --- | --- | --- |
|  |  | **cell vol** | **GR (mu)** | **PICcq** | **PIC prod** | **POCcq** | **POC prod** | **PONcq** | **PIC:POC** | **POC:PON** | **Sr:Ca calcite** | **D-Sr** |
| **coccosphere vol** | **Correlation coef.** | **0.799** | **-0.522** | **0.788** | **0.642** | **0.691** | **0.782** | **0.504** | **0.355** | **0.597** | **0.0262** | **-0.0561** |
|  | P value | 0.000000582 | 0.00519 | 0.00000109 | 0.000304 | 0.0000666 | 0.00000144 | 0.00738 | 0.0691 | 0.00101 | 0.897 | 0.781 |
|  | *No. Samples* | *27* | *27* | *27* | *27* | *27* | *27* | *27* | *27* | *27* | *27* | *27* |
| **cell vol** | **Correlation coef.** | | **-0.479** | **0.847** | **0.742** | **0.725** | **0.84** | **0.583** | **0.302** | **0.47** | **-0.14** | **-0.234** |
|  | P value |  | 0.00205 | 2.54E-08 | 0.0000096 | 1.79E-07 | 2.39E-11 | 0.0000969 | 0.126 | 0.00256 | 0.487 | 0.24 |
|  | *No. Samples* | | *39* | *27* | *27* | *39* | *39* | *39* | *27* | *39* | *27* | *27* |
| **GR (mu)** | **Correlation coef.** | |  | **-0.481** | **-0.196** | **-0.313** | **-0.59** | **-0.111** | **-0.322** | **-0.441** | **-0.221** | **-0.142** |
|  | P value |  |  | 0.0111 | 0.327 | 0.0526 | 0.0000761 | 0.501 | 0.102 | 0.005 | 0.268 | 0.481 |
|  | *No. Samples* | |  | *27* | *27* | *39* | *39* | *39* | *27* | *39* | *27* | *27* |
| **PICcq** | **Correlation coef.** | |  |  | **0.936** | **0.727** | **0.774** | **0.559** | **0.641** | **0.5** | **-0.0801** | **-0.153** |
|  | P value |  |  |  | 7.15E-13 | 0.0000173 | 0.00000215 | 0.00245 | 0.000311 | 0.00789 | 0.691 | 0.446 |
|  | *No. Samples* | |  |  | *27* | *27* | *27* | *27* | *27* | *27* | *27* | *27* |
| **PIC prod** | **Correlation coef.** | |  |  |  | **0.613** | **0.6** | **0.51** | **0.693** | **0.374** | **-0.156** | **-0.206** |
|  | P value |  |  |  |  | 0.000669 | 0.00094 | 0.00662 | 0.000062 | 0.0547 | 0.436 | 0.303 |
|  | *No. Samples* | |  |  |  | *27* | *27* | *27* | *27* | *27* | *27* | *27* |
| **POCcq** | **Correlation coef.** | |  |  |  |  | **0.924** | **0.917** | **-0.00312** | **0.473** | **0.0215** | **-0.0621** |
|  | P value |  |  |  |  |  | 4.92E-17 | 2.66E-16 | 0.988 | 0.00234 | 0.915 | 0.758 |
|  | *No. Samples* | |  |  |  |  | *39* | *39* | *27* | *39* | *27* | *27* |
| **POC prod** | **Correlation coef.** | |  |  |  |  |  | **0.808** | **0.0934** | **0.494** | **0.0152** | **-0.0828** |
|  | P value |  |  |  |  |  |  | 4.81E-10 | 0.643 | 0.00139 | 0.94 | 0.681 |
|  | *No. Samples* | |  |  |  |  |  | *39* | *27* | *39* | *27* | *27* |
| **PONcq** | **Correlation coef.** | |  |  |  |  |  |  | **-0.0874** | **0.107** | **-0.122** | **-0.189** |
|  | P value |  |  |  |  |  |  |  | 0.665 | 0.516 | 0.544 | 0.345 |
|  | *No. Samples* | |  |  |  |  |  |  | *27* | *39* | *27* | *27* |
| **PIC:POC** | **Correlation coef.** | |  |  |  |  |  |  |  | **0.127** | **-0.154** | **-0.167** |
|  | P value |  |  |  |  |  |  |  |  | 0.528 | 0.442 | 0.406 |
|  | *No. Samples* | |  |  |  |  |  |  |  | *27* | *27* | *27* |
| **POC:PON** | **Correlation coef.** | |  |  |  |  |  |  |  |  | **0.407** | **0.36** |
|  | P value |  |  |  |  |  |  |  |  |  | 0.0353 | 0.065 |
|  | *No. Samples* | |  |  |  |  |  |  |  |  | *27* | *27* |
| **Sr:Ca calcite** | **Correlation coef.** | |  |  |  |  |  |  |  |  |  | **0.99** |
|  | P value |  |  |  |  |  |  |  |  |  |  | 1.10E-22 |
|  | *No. Samples* | |  |  |  |  |  |  |  |  |  | *27* |
|  | | | | | | | | |  |  |  |  |
| The pair(s) of variables with positive correlation coefficients and P values below 0.050 tend to increase together. | | | | | | | | |  | | | |
| For the pairs with negative correlation coefficients and P values below 0.050, one variable tends to decrease while the other increases. | | | | | | | | | |  | | |
| For pairs with P values greater than 0.050, there is no significant relationship between the two variables. | | | | | | | |  | | | | |
